# Clinical utility of an evolving cholestasis gene panel in 10,000 children and adults

**DOI:** 10.3389/fped.2026.1841006

**Published:** 2026-05-18

**Authors:** Brett J. Hoskins, Tiziano Pramparo, Ethan Gough, Amy Ponte, Rana Dutta, Wikrom Karnsakul

**Affiliations:** 1 Division of Pediatric Gastroenterology, Hepatology and Nutrition, Department of Pediatrics, Riley Hospital for Children at IU Health, Indiana University School of Medicine, Indianapolis, IN, United States; 2 Mirum Pharmaceuticals Inc., Foster City, CA, United States; 3 Department of International Health, Johns Hopkins Bloomberg School of Public Health, Baltimore, MD, United States; 4 Sanofi, Bridgewater, NJ, United States; 5 Division of Pediatric Gastroenterology, Hepatology and Nutrition, Department of Pediatrics, The Johns Hopkins University School of Medicine, Baltimore, MD, United States

**Keywords:** cholestasis, diagnostic yield, hepatic disease, next-generation sequencing, pediatrics

## Abstract

**Objective:**

Cholestasis has diverse causes, with genetic factors playing a key role. Diagnosis is challenging due to varied presentations and overlapping genetic conditions. Next-generation sequencing cholestasis gene panels enable faster, more accurate identification of genetic causes. This study summarizes results from more than 10,000 tests, highlighting their clinical utility.

**Methods:**

We analyzed aggregate data from a 77-gene cholestasis panel used between 2016 and 2022. Eligible patients had unexplained cholestasis or chronic liver disease. DNA sequencing utilized custom capture libraries (SureSelect 2016–2021 and PGxome® 2021–2022). Variants were classified per ACMG/AMP guidelines. Definitive diagnoses required biallelic pathogenic/likely pathogenic variants in autosomal recessive genes or a single pathogenic/likely pathogenic variant in autosomal dominant *JAG1* or *NOTCH2*. Potential diagnoses involved one pathogenic/likely pathogenic variant in autosomal recessive genes plus one variant of uncertain significance.

**Results:**

Of 10,894 samples analyzed, 51.1% were from patients less than 1 year old and 9.2% from those 18 years of age or older. Overall, 2917 patients carried one or more pathogenic or likely pathogenic variant(s). Diagnostic yield was 6.8% for definitive and 2.2% for potential diagnoses. Definitive findings were most common in *JAG1, SERPINA1, ABCC2*, *ABCB11*, *CFTR*, *POLG*, and *NOTCH2*. Potential diagnoses commonly involved *ABCC2*, *ABCB4, ABCB11, CFTR,* and *PKHD1.* Monoallelic variants were frequent in *SERPINA1*, *CFTR*, *DHCR7*, *ABCB4*, and *PKHD1*.

**Conclusions:**

These cholestasis gene panel results reinforce their value in diagnosing and identifying complex genetic causes of cholestasis, especially in infants less than 1 year old. Early detection supports timely intervention, and the panel provides clearer insight to support accurate diagnoses and inform potential therapeutic strategies.

## 1 Introduction

Cholestasis, defined by impaired bile formation or flow, arises from diverse hepatobiliary disorders with both environmental and genetic causes. In neonatal cholestasis, genetic etiologies account for about one-quarter of cases, reinforcing the importance of early diagnosis (1–3). These include multisystem syndromes like Alagille syndrome (ALGS) (*JAG1* or *NOTCH2* variants) and isolated hepatic conditions such as alpha-1-antitrypsin deficiency (A1ATD) (*SERPINA1*). Other inherited causes include bile acid synthesis disorders (BASD) due to single enzyme defects (*HSD3B7*, *AKR1D1*, *CYP27A1*), progressive familial intrahepatic cholestasis (PFIC)-related canalicular transport defects (*ATP8B1*, *ABCB11*, *ABCB4*, *TJP2*, *NR1H4*, *MYO5B, SLC51A*, *USP53*, *KIF12*, *ZFYVE19*), and systemic disorders such as cystic fibrosis (CF) (*CFTR*) and autosomal recessive polycystic kidney disease (*PKHD1*) (2, 4–9).

Although often considered a neonatal disorder, cholestasis can present in childhood or adulthood, reflecting a lifelong phenotypic expression. For example, *ABCB11* variants are associated with benign recurrent intrahepatic cholestasis (BRIC), and *ABCB4* variants with intrahepatic cholestasis of pregnancy (ICP) and other adult-onset forms. Genetic susceptibility also contributes to complex hepatobiliary diseases, including gallstones, fatty liver, and drug-induced liver injury (DILI), demonstrating the broad influence of inherited factors on hepatic physiology (10–14).

Despite major advances, pinpointing the genetic cause of cholestasis remains challenging. Overlapping phenotypes, variable penetrance, and numerous variants of uncertain significance (VUS) complicate diagnosis and management. Next-generation sequencing (NGS) has improved the diagnostic landscape by enabling simultaneous analysis of many genes implicated in bile acid metabolism, transport, and signaling (8, 15–18).

This study presents a large-scale analysis of cholestasis gene panel testing in children and young adults, offering updated insight into diagnostic yield and the evolving genetic landscape. With a dataset nearly five times larger than earlier reports (8), it refines diagnostic yield estimates and highlights newly implicated genes as well as trends in variant classification. The findings affirm both the clinical utility and current interpretive challenges of broad NGS-based testing in cholestatic disorders.

## 2 Materials and methods

### 2.1 Study design and population

This retrospective analysis evaluated patients with a clinical diagnosis or history of cholestasis who underwent NGS using a targeted cholestasis gene panel. The panel expanded from 57 to 77 genes in 2018–2019 (Supplementary Table 1). Testing was provided at no cost through the Genetic Cholestasis Panel Testing Program, sponsored by Retrophin, Travere Therapeutics, and Mirum Pharmaceuticals, Inc., to facilitate early diagnosis and management of genetic cholestasis.

Eligible patients had intrahepatic cholestasis or chronic liver disease of unknown cause. Exclusions included extrahepatic disorders (e.g., biliary atresia, choledochal cyst, large-duct primary sclerosing cholangitis [PSC]) and cholestasis secondary to total parenteral nutrition. Blood or saliva samples were collected by healthcare providers and sent to Eurofins/Emory Genetics Laboratory (EGL; 2016–2021) and PreventionGenetics (PG; 2021–2022) with a completed test requisition form and signed informed consent. The study was approved by the Institutional Review Board of Johns Hopkins University School of Medicine (IRB00299237) with a waiver of informed consent. All research was conducted in accordance with the Declarations of Helsinki and Istanbul.

### 2.2 Genetic testing and variant classification

DNA was extracted and sequenced using hybrid-capture enrichment (SureSelect [EGL] or PGxome® [PG]) followed by NGS. Sequence reads were aligned to GRCh37/UCSC hg19 reference genome. Variant interpretation followed ACMG/AMP guidelines, and variants were classified as pathogenic (P), likely pathogenic (LP), variant of uncertain significance (VUS), likely benign (LB), or benign (B). Copy number variants (CNVs) were assessed using validated read-depth-based algorithms and are summarized separately in Supplementary Table 2.

### 2.3 Definition of diagnostic categories

A definitive molecular diagnosis was defined as either:

- Two pathogenic/likely pathogenic (P/LP) single nucleotide variants (SNVs; homozygous or compound heterozygous) in an autosomal recessive gene, or
- One P/LP SNV in an autosomal dominant gene (*JAG1* or *NOTCH2* in ALGS).

A potential diagnosis was assigned to cases with one P/LP variant and one VUS in the same autosomal recessive gene. Monoallelic findings in autosomal recessive genes were summarized separately.

Copy number variants were analyzed as part of the testing workflow but were evaluated independently from SNVs and were not incorporated into diagnostic-yield calculations.

The diagnostic yield was calculated as the number of positive results (definitive + potential diagnoses) divided by the total number of completed tests. Demographic metadata (age and ethnicity) were available for all samples, whereas sex data were only available for those analyzed by PG. Clinical data were not available for the present analysis.

### 2.4 Subanalysis of PFIC-associated genes

A targeted subanalysis evaluated heterozygous and compound heterozygous variants in *ATP8B1*, *ABCB11*, *ABCB4*, *TJP2*, *NR1H4*, *MYO5B*, and *SLC51A*—genes implicated in PFIC that frequently generate partial or ambiguous results that complicate clinical interpretation. Other PFIC-associated genes, including *USP53*, *KIF12*, and *ZFYVE19*, were not included in the current panel.

This subanalysis aimed to:

1. Quantify the frequency of monoallelic pathogenic variants in each gene.
2. Identify cases with a potential diagnosis (P/LP + VUS); and
3. Explore possible cross-gene interactions, where monoallelic pathogenic variants in one PFIC gene co-occurred with VUS in another.

Cases meeting these criteria were extracted from the larger dataset and analyzed separately to assess potential digenic or modifier effects.

### 2.5 Enrichment analysis of VUS for genes of interest

To evaluate the significance of VUS in this cohort, their frequency in a clinically enriched cholestatic disorder population was compared with population-based reference data from the Genome Aggregation Database (gnomAD). Heterozygous VUS (as classified by the diagnostic laboratories) were extracted across 11 genes of interest: *JAG1, NOTCH2, ATP8B1, ABCB11, ABCB4, TJP2, NR1H4, MYO5B, HSD3B7, AKR1D1*, and *CYP27A1*. Corresponding variant frequencies from gnomAD v4.1 encompassing all population groups were matched by gene symbol and MANE Select transcript consequence. The number of variants analyzed per gene was 105, 200, 113, 195, 164, 186, 22, 110, 57, 34, and 99, respectively. Statistical comparison between the two groups was conducted using a two-proportion z-test, and *P* values were adjusted for multiple testing using the Benjamini–Hochberg (BH) method. Results were considered statistically significant for adjusted *P* values <0.05.

## 3 Results

### 3.1 Cohort characteristics

A total of 10,894 samples were analyzed between February 2016 and February 2022 across two diagnostic laboratories. Panel size expanded over time: ∼4000 samples used the original 57-gene panel (2/2016–11/2018), ∼4600 the 66-gene panel (11/2018–2019), and ∼2400 the final 77-gene panel (2022), with each sample evaluated using the version active at testing (Table 1 and Supplementary Table 1). Patients ranged in age from birth to 88 years, with infants and young children predominating. Over half (51.1%) were under one year old, while 9.2% were adults. Specific results from the adult subset will be analyzed and reported separately (Table 2). Infants aged 2 to 3 months represented the largest age group (13.0%), followed by 1-year-olds (12.6%).

**Table 1:** Genes included in the cholestasis sequencing panel.

Cholestasis-related genetic disorders	Genes
BASD due to single enzyme defects and CTX	*AKR1D1; AMACR; BAAT; CYP27A1; CYP7A1; CYP7B1; DHCR7; HSD3B7; SLC27A5*
Peroxisomal disorders, including ZSD	*PEX1; PEX10; PEX11B; PEX12; PEX13; PEX14; PEX16; PEX19; PEX2; PEX26; PEX3; PEX5; PEX6; PEX7*
PFIC	*ABCB11; ABCB4; ATP8B1; MYO5B; NR1H4; TJP2*
ALGS	*JAG1; NOTCH2*
A1ATD	*SERPINA1*
CF	*CFTR*
Polycystic kidney disease	*PKHD1*
Other genetic causes of cholestasis	*ABCC2; ABCG5; ABCG8; ACOX2; AKR1C4; ALDOB; CC2D2A; CLDN1; DCDC2; DGUOK; EHHADH; FAH; GNAS; GPBAR1; HNF1B; HSD17B4; INVS; KMT2D; LIPA; MKS1; MPV17; NPC1; NPC2; NPHP1; NPHP3; NPHP4; PKD1L1; POLG; SCP2; SLC10A1; SLC10A2; SLC25A13; SLC51A; SLC51B; SLCO1B3; SMPD1; TALDO1; TMEM216; TRMU; UGT1A1; UTP4; VIPAS39; VPS33B*

A1ATD, alpha-1-antitrypsin deficiency; ALGS, Alagille syndrome; BASD, bile acid synthesis disorders; CF, cystic fibrosis; CTX, cerebrotendinous xanthomatosis; PFIC, progressive familial intrahepatic cholestasis; ZSD, Zellweger spectrum disorders.

**Table 2:** Key demographics and baseline characteristics.

Characteristics	Total (*N* = 10,894)
Age range, y	0–88
Age categories, *n* (%)	
<1 y	5570 (51.1)
1–10 y	2850 (26.1)
11–17 y	1477 (13.6)
>18 y	997 (9.2)
Sex,^a^n (%)	
Female	1281/3275 (39.1)
Male	1994/3275 (60.9)
Ethnicity, *n* (%)	
Caucasian/Northwestern European	3681 (33.8)
Hispanic	2010 (18.5)
African American	1621 (14.9)
Asian	658 (6.0)
Native American	104 (1.0)
Other	122 (1.1)
Unknown	2080 (19.1)
Mixed/multiple	618 (5.7)

a Sex data were only provided by PG. PG, PreventionGenetics.

Among patients with available sex data (*n* = 3275), 60.9% were male and 39.1% female. Ethnicity was reported for all samples. Larger self-identified groups were Caucasian/Northwestern European (33.8%), Hispanic (18.5%), African American (14.9%), and Asian (6.0%). Smaller groups included Native American (1.0%) and Other (1.1%), while 19.1% reported unknown and 5.7% mixed or multiple ethnic backgrounds (Table 2).

### 3.2 Overall variant distribution

**Figure 1:**
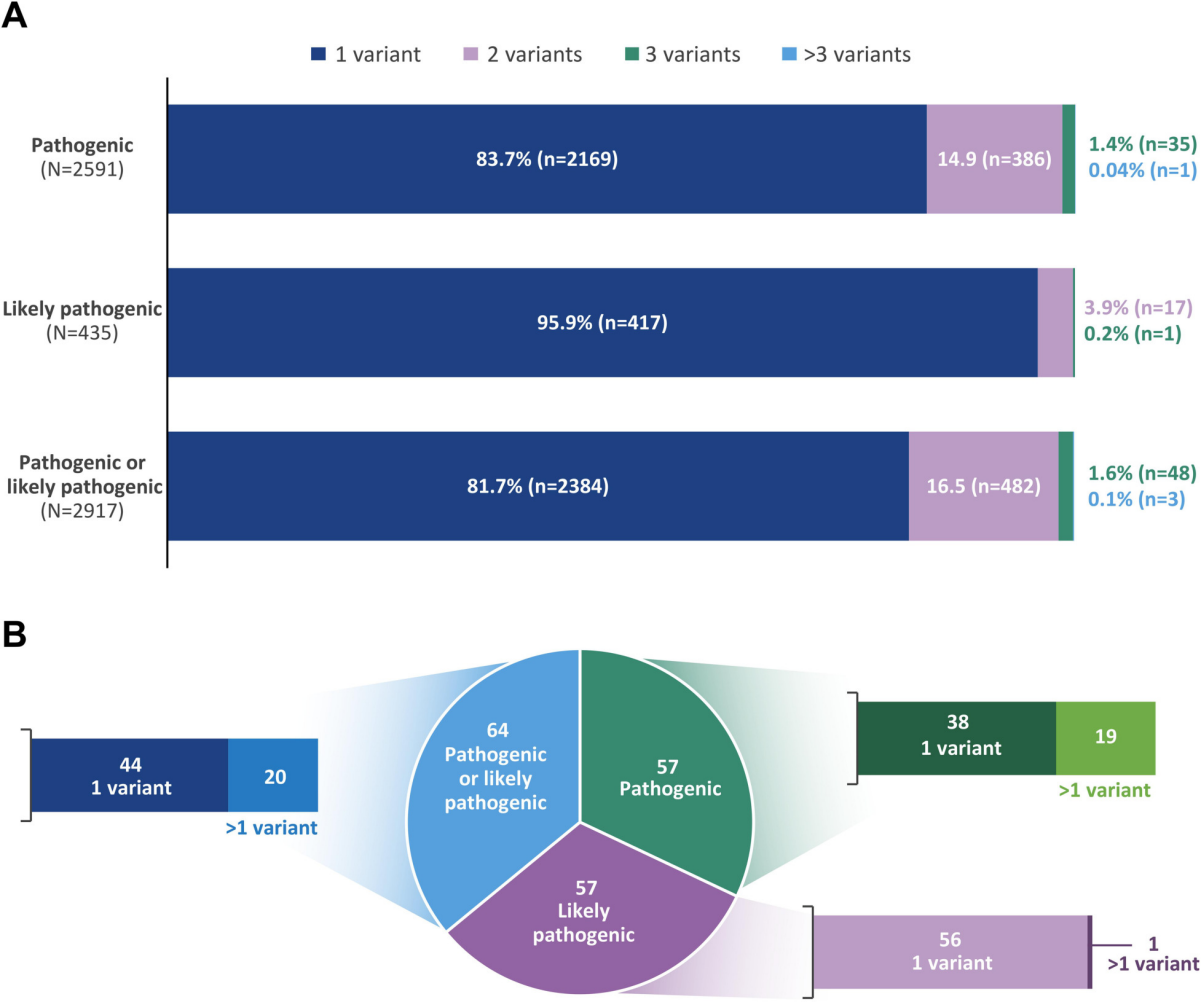
**(A)** distribution of reported pathogenic and likely pathogenic variants in patients with ≥1 variant across genes included in the panel. **(B)** Frequency of genes with pathogenic and likely pathogenic variants.

Across all genes analyzed, 2591 patients had at least one *P* variant and 435 had at least one LP variant, totaling 2917 patients (26.8%) with a reportable P/LP variant. Of these, 81.7% (*n* = 2384) carried a single P/LP variant, 16.5% (*n* = 482) carried two, and 1.6% (*n* = 48) carried three; only three patients (0.1%) harbored more than three reportable variants (Figure 1A).

Overall, *P* variants were identified in 57 genes and LP variants in 57 partially overlapping genes, with a combined total of 64 unique genes harboring at least one *P* or LP variant (Figure 1B).

### 3.3 Copy number variants

Copy number variants were identified in over 50 genes (Supplementary Table 2) but were most frequently observed in *SLCO1B3* (*n* = 26), *SLCO1B1* (*n* = 9), *ABCG5* (*n* = 7), *ABCG8* (*n* = 6), and *ABCC2* (*n* = 6). Several CNVs were classified as *P* or LP, including recurrent deletions in *NPHP1*, *NPHP4*, *HNF1B* (as part of multigene events), *ABCB11*, *ATP8B1*, *AMACR*, and *SLC51A*. The majority of CNVs were VUS, most prominently involving *ABCG5*, *ABCG8*, *PEX11B*, *DCDC2*, and *SCP2*.

### 3.4 Diagnostic yield

**Figure 2:**
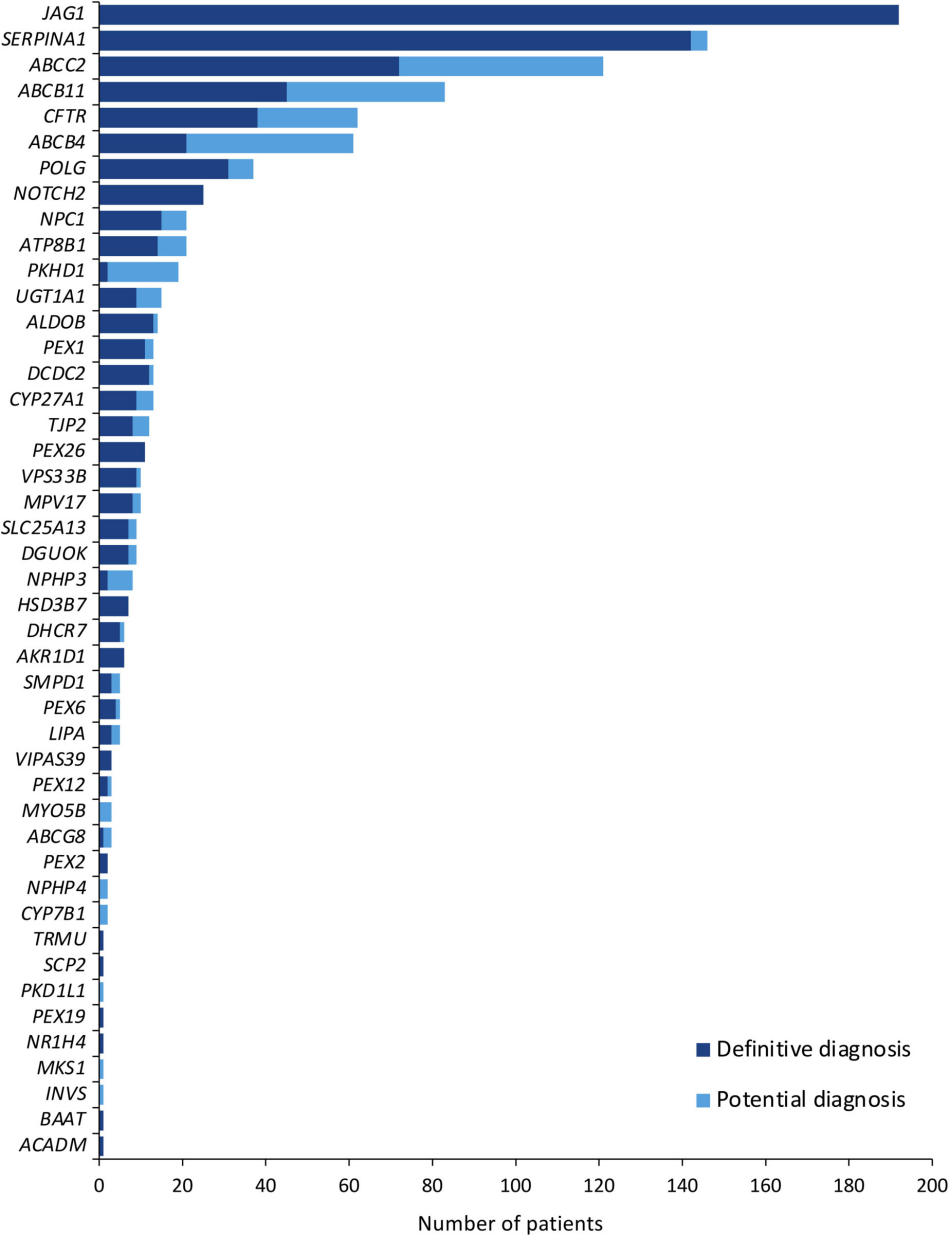
Frequency of patients with >1 pathogenic and/or likely pathogenic variant with definitive and potential diagnoses.

Based on variant classification and zygosity, 745 patients (6.8%) met the criteria for a definitive molecular diagnosis, while 241 (2.2%) had potential diagnoses, with an overall diagnostic yield of 9.0% (Table 3). The remainder of the cohort had nondiagnostic findings, which encompassed VUS, B and LB variants, reduced function or at-risk alleles, and negative genetic results (Table 4). Definitive diagnoses were most frequently attributed to *JAG1* (*n* = 192), *SERPINA1* (*n* = 142), *ABCC2* (*n* = 72), *ABCB11* (*n* = 45), *CFTR* (*n* = 38), *POLG* (*n* = 31), and *NOTCH2* (*n* = 25). Potential diagnoses most often involved *ABCC2* (*n* = 49), *ABCB4* (*n* = 40), *ABCB11* (*n* = 38), *CFTR* (*n* = 24), and *PKHD1* (*n* = 17) (Table 3; Figure 2).

**Table 3:** Frequency of patients with >1 P and/or LP variant with definitive and potential diagnoses and monoallelic variants^a^.

Gene^a^	Definite diagnosis	Potential diagnosis	Monoallelic variants
*ABCB11*	45	38	81
*ABCB4*	21	40	100
*ABCC2*	72	49	79
*ABCG5*			9
*ABCG8*	1	2	40
*ACADM*	1		
*ACOX2*			6
*AKR1D1*	6		14
*ALDOB*	13	1	83
*AMACR*			8
*ATP8B1*	14	7	22
*BAAT*	1		
*CC2D2A*			31
*CFTR*	38	24	360
*CYP27A1*	9	4	65
*CYP7B1*		2	24
*DCDC2*	12	1	4
*DGUOK*	7	2	10
*DHCR7*	5	1	157
*FAH*			9
*GNAS*			1
*HNF1B*			10
*HSD17B4*			10
*HSD3B7*	7		5
*INVS*		1	11
*JAG1*	192		
*KMT2D*			4
*LIPA*	3	2	33
*MKS1*		1	27
*MPV17*	8	2	16
*MYO5B*		3	5
*NOTCH2*	25		
*NPC1*	15	6	34
*NPC2*			5
*NPHP1*			11
*NPHP3*	2	6	28
*NPHP4*		2	25
*NR1H4*	1		6
*PEX1*	11	2	32
*PEX10*			11
*PEX11B*			3
*PEX12*	2	1	11
*PEX19*	1		1
*PEX2*	2		6
*PEX26*	11		5
*PEX3*			4
*PEX5*			6
*PEX6*	4	1	8
*PEX7*			21
*PKD1L1*		1	10
*PKHD1*	2	17	89
*POLG*	31	6	71
*SCP2*	1		3
*SERPINA1*	142	4	569
*SLC10A1*			5
*SLC25A13*	7	2	18
*SMPD1*	3	2	39
*TALDO1*			2
*TJP2*	8	4	10
*TMEM216*			4
*TRMU*	1		4
*UGT1A1*	9	6	36
*VIPAS39*	3		6
*VPS33B*	9	1	10

LP, likely pathogenic; P, pathogenic.

a The following genes from the 77 gene panel had no P or LP variants: *AKR1C4, CLDN1, CYP7A1, EHHADH, GPBAR1, PEX13, PEX14, PEX16, SLC10A2, SLC27A5, SLC51A, SLC51B, SLCO1B3, UTP4*.

**Table 4:** Combinations of P variants, LP variants, and VUS of interest .

Gene	HOM P/LP, n	Two alleles P/LP, n	Two alleles 1 VUS + 1 P/LP, n	One allele P/LP, n	HOM VUS, n	1 or 2 HET alleles VUS, n	Other reportable, n	B/LB, n	Negative sample, n
*ABCB11* ^a^	21	24	38	81	14	459	11		33
*ABCB4* ^a^	14	7	40	100	25	487		1	30
*ABCC2* ^a^	30	42	49	79	21	582		2	26
*ABCG5* ^a^				9		296			14
*ABCG8* ^a^		1	2	40	2	381		8	31
*ACADM* ^b^	1								
*ACOX2* ^a^				6		75			
*AKR1C4*						53			
*AKR1D1*	6			14	1	72			7
*ALDOB* ^a^	7	6	1	83	1	201		2	5
*AMACR*				8		99			6
*ATP8B1* ^a^	10	4	7	22	5	327		5	26
*BAAT* ^a^	1					99			7
*CC2D2A* ^a^				31	3	411		6	25
*CFTR* ^a^	19	19	24	360	7	973	668	67	36
*CLDN1*					1	41			6
*CYP27A1* ^a^	7	2	4	65	3	257			7
*CYP7A1* ^a^					1	166			8
*CYP7B1* ^a^			2	24	1	133		1	7
*DCDC2* ^a^	9	3	1	4	2	111		13	3
*DGUOK* ^a^	3	4	2	10	4	138			10
*DHCR7* ^a^		5	1	157	1	172		4	12
*DNAH11* ^b^						1			
*DNAH6* ^b^						1			
*EHHADH* ^a^					1	251			8
*FAH* ^a^				9	2	90	11	90	36
*FOXJ1* ^b^						1			
*GNAS*				1		84			
*GPBAR1*					1	157			8
*HNF1B* ^a^				10		118			7
*HSD17B4*				10	2	179		1	10
*HSD3B7* ^a^	7			5	3	132			6
*INVS* ^a^			1	11	1	265			13
*JAG1*				192	1	252		3	26
*KMT2D* ^a^				4	1	255			
*LIPA* ^a^	1	2	2	33	1	96		3	13
*MKS1* ^a^			1	27		276		1	21
*MMP21* ^b^						1			
*MPV17*	8		2	16	3	31			6
*MYO5B* ^a^			3	5	1	141			
*NOTCH2* ^a^				25	1	364		9	29
*NPC1* ^a^	8	7	6	34	6	310		2	28
*NPC2*				5		105			
*NPHP1* ^a^				11	1	168		9	9
*NPHP3* ^a^		2	6	28	1	291		1	17
*NPHP4* ^a^			2	25	3	786		4	60
*NR1H4* ^a^	1			6		108			7
*PEX1* ^a^	4	7	2	32	1	268		1	8
*PEX10* ^a^				11		158		14	7
*PEX11B*				3		60			7
*PEX12*		2	1	11		92			3
*PEX13* ^a^						92			4
*PEX14* ^a^						138			12
*PEX16*						86			10
*PEX19* ^a^		1		1	4	105		1	9
*PEX2* ^a^	1	1		6		88		1	5
*PEX26* ^a^	10	1		5	1	129			12
*PEX3* ^a^				4		48			6
*PEX5* ^a^				6	2	179		3	9
*PEX6* ^a^	3	1	1	8	3	232	51	2	14
*PEX7*				21		63			8
*PKD1L1* ^a^			1	10	1	180			
*PKHD1* ^a^	1	1	17	89	8	1136		15	68
*POLG* ^a^		31	6	71	1	389		18	30
*SCP2* ^a^	1			3		76			10
*SERPINA1* ^a^	136	6	4	569		205	482		16
*SLC10A1* ^a^				5	4	234		3	11
*SLC10A2* ^a^					3	307			20
*SLC25A13* ^a^	3	4	2	18		187			7
*SLC27A5* ^a^					2	242		18	23
*SLC51A*						25			
*SLC51B*						6			
*SLCO1B3* ^a^					2	88			
*SMPD1* ^a^	2	1	2	39	1	257		5	30
*TALDO1*				2		30			
*TJP2* ^a^	7	1	4	10	8	386		12	22
*TMEM216*				4		51			3
*TRMU* ^a^	1			4	1	139			8
*UGT1A1* ^a^	6	3	6	36	10	250	94	3	15
*UTP4*						42			
*VIPAS39* ^a^	2	1		6	1	128		2	14
*VPS33B* ^a^	6	3	1	10	3	161			11
All Genes of Interest	336	192	241	2534	177	16253	1317	330	1005

Reduced function/at risk variants were identified in CTFR (*n* = 246) and UGT1A1 (*n* = 1895).

B, benign; CNV, copy number variant; HET, heterozygous; HOM, homozygous; LB, likely benign; LP, likely pathogenic; P, pathogenic; VUS, variant of uncertain significance.

a Two or more VUS.

b Genes from the CNV analysis.

### 3.5 Gene-level variant combinations and monoallelic findings

Genotype combination analysis related to definitive diagnoses revealed 336 patients homozygous for a P/LP variant and 192 compound heterozygotes carrying two P/LP alleles (Table 4). The most common contributors to this group were *SERPINA1* (*n* = 136 homozygous, 6 compound heterozygous), *ABCC2* (*n* = 30 and 42, respectively), *ABCB11* (*n* = 21 and 24), *CFTR* (*n* = 19 and 19), and *POLG* (*n* = 0 and 31). Among the 241 patients with a potential diagnosis (one P/LP plus one VUS), the most frequent genes were *ABCC2* (*n* = 49), *ABCB4* (*n* = 40), *ABCB11* (*n* = 38), and *CFTR* (*n* = 24).

Monoallelic findings were common in *SERPINA1* (*n* = 569), *CFTR* (*n* = 360), *DHCR7* (*n* = 157), *ABCB4* (*n* = 100), and *PKHD1* (*n* = 89), suggesting potential oligogenic or modifier roles in combination with other genotypes. A PFIC gene subanalysis is provided below.

### 3.6 Variants of uncertain significance and reduced-function alleles

VUS were widespread, affecting over 16,000 total alleles across genes of interest. High counts of heterozygous VUS occurred in *PKHD1* (*n* = 1136), *CFTR* (*n* = 973), *ABCC2* (*n* = 582), *ABCB4* (*n* = 487), *ABCB11* (*n* = 459) (Table 4). Autosomal dominant *JAG1 and NOTCH2* carried 252 and 364 heterozygous VUS, respectively. Homozygous VUS were most common in *ABCB4* (*n* = 25), *ABCC2* (*n* = 21), *ABCB11* (*n* = 14), and *UGT1A1* (*n* = 10).

Comparing heterozygous VUS frequencies across 11 cholestasis-related genes revealed major differences between our clinically enriched cholestasis cohort and the population reference dataset (gnomAD v4.1) with a significantly higher frequency (BH-adjusted *P* < 0.05) in most genes, including *JAG1, NOTCH2, ABCB11, ABCB4, TJP2, HSD3B7, AKR1D1*, and *CYP27A1* (Supplementary Table 3). The absolute differences in frequency ranged from 0.004 to 0.024, with confidence intervals indicating robust separation from the reference population. *NR1H4* displayed a lower VUS frequency in the cholestasis cohort relative to gnomAD (diff = −0.006, BH-adjusted *P* < 0.001), and *MYO5B* showed no significant difference (diff = 0.001, BH-adjusted *P* = 0.332). These findings suggest that VUS in key cholestasis genes are enriched in a clinically affected population compared with a general population, supporting their potential disease relevance.

Genes with a high proportion of reduced-function or “at-risk” variants included *UGT1A1* (*n* = 1895) and *CFTR* (*n* = 246) (Table 4). Some genes had findings of “other reportable,” including *SERPINA1* (*n* = 482), *CFTR* (*n* = 668), *UGT1A1* (*n* = 94), and *PEX6* (*n* = 51), which represent secondary variants that are clinically significant but less likely to be a primary cause of disease.

### 3.7 PFIC-associated genes

Monoallelic P/LP variants were detected in six PFIC-associated genes: *ABCB4* (*n* = 100), *ABCB11* (*n* = 81), *ATP8B1* (*n* = 22), *TJP2* (*n* = 10), *NR1H4* (*n* = 6), and *MYO5B* (*n* = 5); none were found in *SLC51A* (Tables 3, 4). Potential diagnoses involving a pathogenic variant plus a VUS occurred in *ABCB4* (40 cases), *ABCB11* (38 cases), *ATP8B1* (7 cases), *TJP2* (4 cases), and *MYO5B* (3 cases). No patients carried monoallelic pathogenic variants in multiple PFIC genes, though VUS in other genes were occasionally identified.

Among *ATP8B1* monoallelic P/LP variants (*n* = 22), only one also harbored an *ABCB4* VUS. For *ABCB11* monoallelic P/LP variants (*n* = 81), overlapping VUS occurred in *ATP8B1* (1 case), *ABCB4* (1 case), *TJP2* (3 cases), *NR1H4* (1 case), and *MYO5B* (2 cases). Among *ABCB4* monoallelic P/LP variants (*n* = 100), VUS appeared in *ATP8B1* (4 cases), *ABCB11* (2 cases), *TJP2* (1 case), *NR1H4* (2 cases), and *MYO5B* (2 cases).

In *TJP2* monoallelic P/LP variants (*n* = 10), one had a *ATP8B1* VUS and three had *ABCB4* VUS. For *NR1H4* monoallelic P/LP variants (*n* = 6), two had *ABCB11* VUS and one had a *TJP2* VUS. These findings highlight the complex interplay between pathogenic variants and VUS in PFIC-associated genes, reinforcing the importance of comprehensive genetic evaluation in cholestasis.

### 3.8 *SERPINA1* variant subtypes

**Figure 3:**
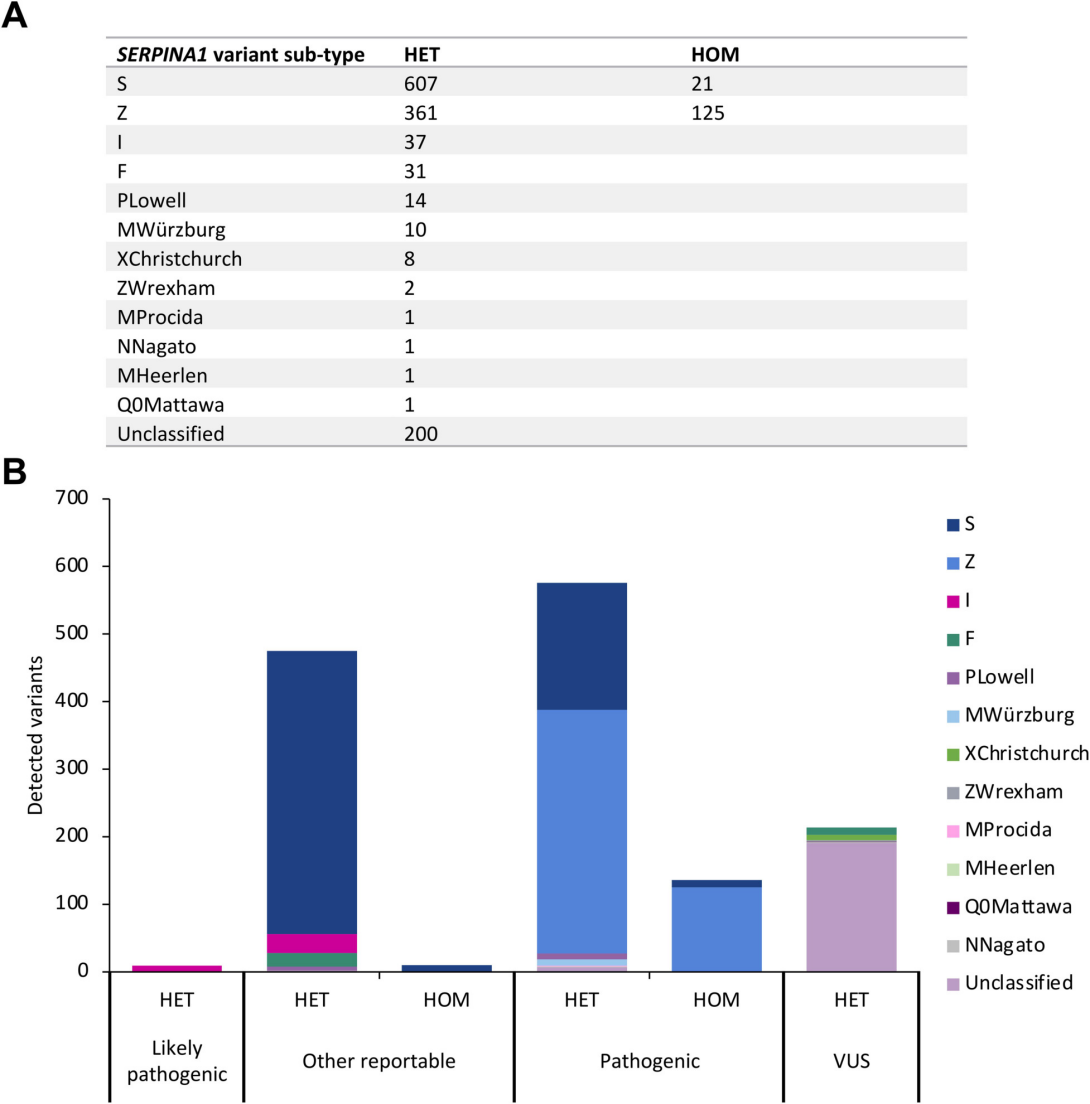
**(A)** distribution of heterozygous and homozygous *SERPINA1* variant subtypes. **(B)** Pathogenicity classification of *SERPINA1* variant subtypes detected in gene panel screening. HET, heterozygous; HOM, homozygous; VUS, variant of uncertain significance.

*SERPINA1* variant analysis revealed a predominance of the common S and Z alleles in heterozygous and homozygous genotypes (Figure 3A). The S variant was detected in 607 heterozygous and 21 homozygous patients, and the Z variant in 361 heterozygous and 125 homozygous patients. Twelve patients were S/Z compound heterozygotes. Other *SERPINA1* variants were less common, including I (*n* = 37), F (*n* = 31), PLowell (*n* = 14), MWürzburg (*n* = 10), XChristchurch (*n* = 8), ZWrexham (*n* = 2), and single instances of MProcida, NNagato, MHeerlen, and Q0Mattawa (19). An additional 200 samples had an unclassified *SERPINA1* variant (Figure 3B). Overall, S and Z alleles accounted for the majority of detected variants, consistent with their known predominance in A1ATD–related genotypes.

### 3.9 Summary of gene contribution

Among definitive diagnoses, *JAG1* and *SERPINA1* together accounted for nearly half of cases, while *ABCC2*, *ABCB4*, *ABCB11*, and *CFTR* collectively represented approximately one-quarter. Smaller contributions were observed for *POLG* and *NOTCH2*.

## 4 Discussion

This study represents the largest evaluation to date of comprehensive genetic testing for cholestasis, encompassing more than 10,000 patients across the pediatric–adult age spectrum. The scale of the cohort allows refinement of diagnostic yield estimates, deeper characterization of variant patterns, and a clearer understanding of the interpretive challenges inherent to cholestasis genomics. Several consistent themes emerged: (1) a modest but clinically meaningful diagnostic yield, particularly in infants; (2) a high burden of monoallelic pathogenic variants and VUS across recessive genes; (3) complex interpretation within PFIC-associated transport genes; and (4) evidence for modifier or oligogenic contributions to cholestatic phenotypes. Collectively, these findings highlight both the utility and limitations of panel-based testing and provide direction for the next phase of precision diagnostics in cholestatic liver disease.

Comprehensive cholestasis panel testing identified a definitive or probable molecular diagnosis in ∼9% of patients, most of whom were infants. *JAG1* and *SERPINA1* were leading contributors, with substantial roles for bile acid transport genes (*ABCB4*, *ABCB11*, *ABCC2*) and *CFTR*. Frequent monoallelic findings and P/LP + VUS combinations across recessive loci underscore interpretive complexity of variants and suggest possible modifier, oligogenic, or digenic effects. These patterns emphasize the need for careful genotype review, segregation analysis, and periodic variant reclassification as new functional and population data emerge.

### 4.1 Diagnostic yield and clinical utility of broad NGS panels

Over six years, cholestasis gene panel testing has yielded key insights into a diverse and variably expressed condition. The overall diagnostic yield of 9.0% (6.8% definitive, 2.2% potential) is slightly lower than that previously reported for smaller cohorts (12% yield from 2171 samples [8% definitive, 4% potential]) (8). Several factors likely contributed. First, the panel expanded from 57 to 77 genes, incorporating loci with lower prior evidence and naturally yielding proportionally more VUS. Second, clinical practice around test ordering appears to have evolved over time to include patients with less specific phenotypes, likely diluting yield compared with earlier cohorts enriched for severe neonatal cholestasis. Third, variant classification criteria continue to evolve, with laboratories becoming increasingly conservative in assigning pathogenicity—particularly for missense variants in transporters and signaling proteins.

Despite these constraints, the yield remains clinically impactful. Most definitive diagnoses occurred in infants <1 year old, highlighting the importance of early genetic testing given narrow therapeutic windows in diseases such as ALGS and PFIC.

The predominance of *JAG1*, *NOTCH2*, *SERPINA1*, and canalicular transporter genes aligns with known epidemiology of pediatric cholestasis and confirms that broad panels reliably capture classical monogenic etiologies. Test turnaround has decreased from 3 months to 3 to 4 weeks, with further reduction expected to enhance outcomes and healthcare efficiency. Equally important, negative panel results may help rule out genetic causes, avoid unnecessary invasive diagnostic procedures (e.g., diagnostic evaluation for biliary atresia), and expedite specific management. With the emergence of targeted therapies like ileal bile acid transporter (IBAT) inhibitors, timely diagnosis is increasingly critical for ALGS and PFIC.

### 4.2 Genes with established diagnostic relevance

In our cohort, definitive diagnoses most commonly involved *JAG1*, *SERPINA1*, *ABCC2*, *POLG*, *NOTCH2*, and *ABCB11*. *JAG1* and *NOTCH2* alone accounted for nearly one-third of all definitive diagnoses, reaffirming their central role in ALGS. These two genes represent the most common monogenic causes of pediatric cholestasis in this cohort, underscoring their value in early diagnostic and therapeutic planning.

Canalicular transport genes such as *ATP8B1*, *ABCB11*, and *ABCB4* also contributed to diagnoses but posed substantial interpretive challenges. For *ATP8B1,* the largest published cohort to date excluded most individuals (520 of 759) because their genotypes were noninformative, underscoring the gene's complexity (20). Even homozygous or truncating variants showed poor genotype-phenotype correlations, and neither genotype nor the number of truncating mutations predicted bile acid response or long-term native liver survival. For rare compound heterozygotes, genotype alone was insufficient to assign causality. Instead, serum bile acid levels at presentation and post-surgical biliary diversion showed stronger prognostic outcome, highlighting the need to integrate biochemical data with genotype rather than relying on sequence changes alone (20).

Similarly, *ABCB11* and *ABCB4* harbor numerous missense variants with highly variable functional consequences. *ABCB11* variants span a spectrum from severe PFIC2 to BRIC2 and ICP, with many alleles lacking robust functional annotation (21).

*ABCB4* variants show wide phenotypic variability, ranging from classic PFIC3 to intermittent pediatric cholestasis, ICP, and adult cholestatic or DILI (22–24). Although biallelic pathogenic variants cause classic PFIC3, most reported variants are missense and produce variable severity. In 108 PFIC3 cases, Sharabati et al. noted that many patients identified in adolescence or adulthood, often required liver transplantation later in life (25). Wang et al. likewise identified 118 genetically confirmed PFIC3 cases, of whom 76.9% harbored missense mutations, with exon 7 and 10 hotspots associated with earlier disease onset (26). Severe phenotypes typically involved biallelic or complex heterozygous variants, whereas monoallelic variants or VUS were linked to milder phenotypes such as intermittent pediatric cholestasis, ICP, or adult DILI. Functional studies indicate many variants retain partial multidrug resistance protein 3 (MDR3) activity, explaining delayed progression and ursodeoxycholic acid responsiveness, as well and preserved MDR3 staining on biopsy.

In our cohort, *ABCB4* monoallelic variants and VUS were common, but pathogenicity is unclear when early-life disease is mild or absent, suggesting roles for environmental or genetic modifiers. Improved classification of these genes will require functional studies including transport assays, splicing analysis, and structural modeling, paired with detailed phenotyping. Until then, management of *ABCB4* VUS in children with unexplained cholestasis should remain cautious, individualized, and guided by evidence.

### 4.3 Genes with emerging or modifier roles

Classic causes of neonatal cholestasis such as CF and A1ATD were rarely detected in biallelic form, likely reflecting prior targeted testing. However, monoallelic variants in *CFTR*, *SERPINA1*, and *ABCB4* were common, supporting a role of disease modifiers. *CFTR* variants can cause cholestasis, and heterozygosity for the *SERPINA1* Z allele increases liver disease risk in CF by up to fivefold (27, 28). Monoallelic *ABCB4* variants have also been linked to faster progression in PSC and ICP, further supporting polygenic contributions in some pediatric cholestasis cases (29, 30).

*ABCC2* variants encoding the MRP2 transporter for conjugated bilirubin and organic anion excretion were also frequent. While biallelic *ABCC2* variants classically cause Dubin-Johnson syndrome, a benign isolated conjugated hyperbilirubinemia rather than true cholestasis, our data and prior reports suggest broader relevance of *ABCC2* variation in cholestasis susceptibility. Monoallelic and compound heterozygous *ABCC2* genotypes may contribute to transient neonatal cholestasis, DILI susceptibility, and other cholestatic states, particularly in the absence of additional genetic or environmental stressors (31–34).

A subset of patients carried single pathogenic or likely pathogenic variants in bile acid synthesis disorder genes (*CYP27A1*, *DHCR7*, and *AKR1D1)*. Although these do not fulfil criteria for autosomal recessive disease, they raise suspicion for undetected second hits, oligogenic inheritance, or an epigenetic modifier. Given the availability of targeted therapies such as chenodeoxycholic acid and cholic acid, follow-up biochemical testing and, when appropriate, expanded genomic analyses are warranted to clarify significance.

Monoallelic pathogenic variants in peroxisomal genes (*PEX1*, *PEX6*, and *PEX26)* were also common. While biallelic *PEX* variants cause classic Zellweger spectrum disorders (ZSD), single pathogenic alleles raise diagnostic uncertainty. Structural variants, deep intronic changes, or oligogenic combinations may be missed by standard panels, and emerging data support digenic or polygenic inheritance in milder peroxisomal phenotypes such as digenic combinations of *PEX1* and *PEX6* (35). These findings suggest that heterozygous PEX variants may contribute to partial peroxisomal dysfunction that is relevant to early liver disease or neurodevelopmental issues without fulfilling criteria for classic ZSD. In such cases, biochemical testing (e.g., very long-chain fatty acids, bile acid intermediates) and selective use of liver biopsy with electron microscopy can help determine pathogenic relevance, though biopsy should be reserved for unresolved cases given its invasiveness and limited sensitivity.

### 4.4 Challenges in variant interpretation

Interpreting VUS remains a major challenge in cholestasis genetics, especially in *JAG1* and *NOTCH2*. Among patients with clinically diagnosed ALGS, 87% to 94% harbor pathogenic variants in *JAG1*, most commonly truncating changes, while 1% to 2.5% have pathogenic *NOTCH2* variants, typically missense, and 3% to 5% lack a molecular diagnosis (36–39). Many missense *NOTCH2* variants and nontruncating *JAG1* variants remain VUS, complicating clinical interpretation and management. This reflects broader limitations in current databases: approximately 40% of ClinVar entries are VUS or carry conflicting interpretations, and VUS rates have risen sharply since 2020 (40).

Recent work has begun to address these gaps. Functional data remain limited, especially for transmembrane proteins like *JAG1*, where up to 85% of missense variants are unclassified. *NOTCH2* has fewer reported variants but is similarly dominated by missense changes, and its disease mechanisms are less characterized (37). Ongoing reclassification efforts continue to highlight these interpretive limitations In the Global ALagille Alliance (GALA) study cohort, Vandriel et al. evaluated 30 *NOTCH2* variants (including 18 novel) and found that 31% of previously pathogenic or likely pathogenic variants were downgraded to VUS (36). They also identified distinct phenotypic differences between *JAG1*- and *NOTCH2*-related ALGS, with *NOTCH2* variants having fewer classic features. These findings suggest classical phenotyping may miss *NOTCH2*-related disease and support broader testing and continued variant reevaluation.

Scalable functional assays—especially those assessing membrane expression—offer promise for VUS reclassification. In a recent study, 27 of 144 *JAG1* VUS showed abnormal membrane expression, enabling reclassification of 26 (18.1%) as likely pathogenic. Variants in exons 1–7, which encode domains critical for membrane trafficking and *NOTCH2* interaction, are more likely to disrupt function. Loss of cysteine, leucine, or isoleucine in EGF-like domains often causes intracellular retention and loss of function, while gains of proline, lysine, arginine, or serine disrupt protein folding and membrane localization (41). For instance, *JAG1* variants p.Cys271Arg, p.Cys693Tyr, and p.Cys714Tyr can reduce membrane expression and cause perinuclear retention, potentially disrupting protein trafficking (37). In contrast, variants in more tolerant regions (e.g., amino acids 57–73) are less likely pathogenic (41).

Normal trafficking does not exclude dysfunction, as some variants may impair *NOTCH2* binding or downstream signaling despite intact membrane expression. Most pathogenic *NOTCH2* variants are missense (68%) and cluster in exons 7–8 (EGF-like repeats) and exons 31–32 (ANK domain), which account for 85% of reported missense changes (37). Although the current panel provides 99.5% coverage of *JAG1* coding exons, *NOTCH2* exons 1–4 are excluded due to technical limitations. No pathogenic variants have been reported in these regions to date.

These findings emphasize the importance of domain-level and amino acid–specific analysis when interpreting VUS in ALGS-associated genes. Integrating functional data into ACMG/AMP frameworks will be critical for improving diagnostic accuracy and enabling timely clinical decisions.

### 4.5 Toward a polygenic and precision framework

Although no patients in our cohort carried multiple pathogenic variants in PFIC genes, monoallelic pathogenic variants frequently co-occurred with VUS in related genes, suggesting possible modifier effects, compound heterozygosity, or digenic inheritance. For example, *ABCB11* pathogenic variants frequently appeared alongside VUS in *ATP8B1, TJP2*, or *NR1H4*. Similarly digenic combinations among *ATP8B1, ABCB11*, *ABCB4*, and *TJP2* have been reported, supporting gene-gene interactions in cholestasis even without biallelic mutations (42, 43). Functional studies will be essential to validate these interactions and refine variant classification.

Broader genetic dynamics may also shape variant persistence and disease expression (44, 45). Founder effects, genetic drift, and reduced selective pressure (partly due to medically improved survival) may allow deleterious alleles to persist in the population. As more individuals with previously fatal liver diseases survive to reproductive age, variants once removed by natural selection may become more common. These trends, alongside expanding precision medicine, highlight the need for periodic VUS reevaluation and variant interpretation models that consider non-Mendelian mechanisms.

*UGT1A1* illustrates how genes not traditionally linked to monogenic cholestasis may still modify disease risk or treatment response. As a key enzyme in bilirubin and bile acid glucuronidation, *UGT1A1* facilitates detoxification and excretion, and monoallelic variants were common in our cohort. Prior studies support a modifier role; Weismüller et al. linked *UGT1A1* variants to disease expression in PSC (46). Pharmacologic activation of *UGT1A1*, such as with peroxisome proliferator-activated receptor alpha agonists like fenofibrate, can enhance glucuronidation and improve cholestatic outcomes. These findings warrant further investigation of *UGT1A1* as both a disease modifier and therapeutic target.

Over the past six years, panel-based genetic testing has transformed the evaluation of pediatric cholestasis, particularly in infants, where early detection impacts outcomes. The PFIC spectrum now includes *ATP8B1*, *ABCB11*, *ABCB4*, *TJP2*, *NR1H4*, *MYO5B, SLC51A*, *USP53*, *KIF12*, and *ZFYVE19*, marking a shift toward gene-based rather than clinical classification. Our cohort revealed frequent co-occurrence of monoallelic pathogenic variants with VUS, reinforcing the importance of reclassification and functional validation. As evidence for digenic and polygenic contributions grows, expanded panels including emerging cholestatic genes may improve diagnostic yield, cost-effectiveness, counseling, and therapeutic planning. Continued genotype–phenotype correlation studies, even in partially explained cases, remain critical for refining diagnosis and care.

Clinicians currently lack reliable real-time methods to interpret VUS in cholestasis. Reclassification depends on diagnostic laboratories, with no standardized system, prioritization framework, or sustainable funding to support systematic reevaluation. Consequently, the burden of VUS remains high for patients and providers, reflecting a need for rapid and actionable reclassification processes.

### 4.6 Recommendations and limitations

Several limitations affect our study. Many VUS lack functional or segregation data, limiting interpretability, and classical phenotype data were inconsistently available, hindering genotype-phenotype correlation. Variant classifications were based on a single laboratory's framework, affecting generalizability and highlighting the need for harmonized interpretation and shared databases. To address these gaps, we are leading an independent multicenter consortium study to systematically collect clinical and biochemical data, enabling stronger genotype–phenotype correlations and supporting future reclassification efforts. Another limitation of this study is the absence of patient-level clinical data, which precluded subgroup analyses by age, clinical presentation, and phenotype. Because results were provided in aggregate, deidentified form by two independent diagnostic laboratories, genotype–phenotype correlations could not be assessed. Future prospective and multicenter studies integrating detailed clinical, biochemical, and genomic data will be essential to better define these relationships and refine precision diagnostic approaches in cholestatic liver disease.

**Figure 4:**
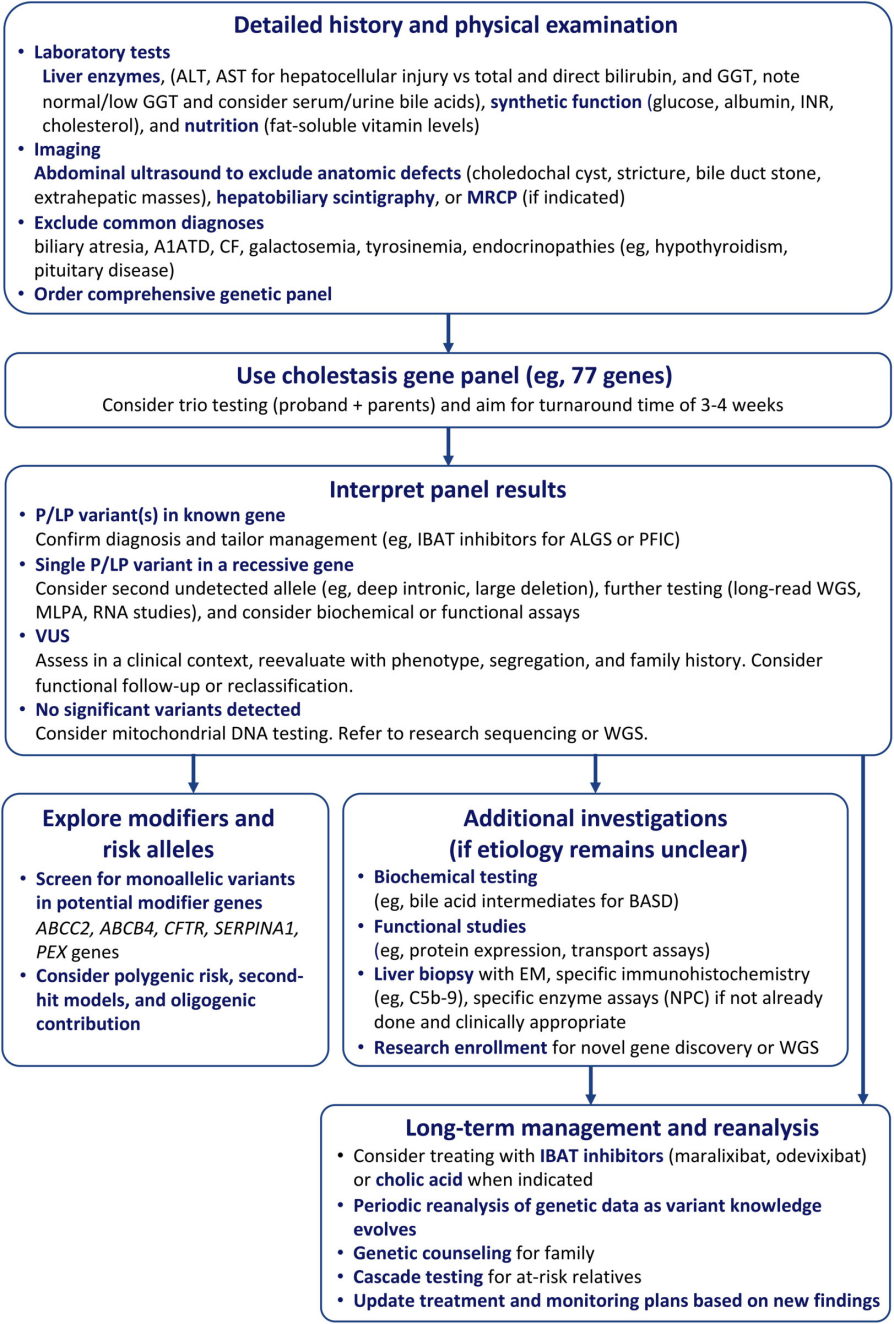
This flowchart outlines a stepwise diagnostic approach to evaluating unexplained neonatal cholestasis (non-bile acid), after biliary atresia is excluded. Key decision points include syndromic features, GGT and bile acid levels, liver biopsy findings, and MRCP to assess for structural anomalies. Genetic/metabolic testing is guided by clinical suspicion, with further evaluation including gene panels, whole exome/genome sequencing, and VUS analysis. Multisystem involvement or inconclusive biopsy may prompt additional testing (e.g., EM, enzyme assays, mitochondrial studies). This algorithm supports a comprehensive yet targeted approach to identifying genetic and metabolic causes of cholestasis. A1ATD, alpha-1-antitrypsin deficiency; ALGS, alagille syndrome; BASD, bile acid synthesis disorder; CF, cystic fibrosis; IBAT, ileal bile acid transporter; INR, international normalized ratio; LP, likely pathogenic; MLPA, multiplex ligation-dependent probe amplification; MRCP, magnetic resonance cholangiopancreatography; NPC, Niemann-Pick type C; P, pathogenic; PFIC, progressive familial intrahepatic cholestasis; VUS, variant of uncertain significance; WGS, whole genome sequencing.

Practical considerations also remain for clinicians evaluating infants with unresolved cholestasis. We propose a tiered diagnostic approach (Figure 4): after excluding common, treatable causes, imaging (e.g., magnetic resonance cholangiopancreatography [MRCP]) and histology (e.g., liver biopsy) can guide further evaluation. Early panel-based genetic testing reduces diagnostic delays and may limit invasive procedures, though VUS can add uncertainty. For patients with persistent cholestasis and negative panel results but syndromic features suggestive of specific disorders, management should proceed based on the most likely diagnosis.

For instance, IBAT inhibitors may benefit patients with ALGS-like features and pruritus, while cholic acid is appropriate in suspected BASD, particularly with abnormal urinary bile acids. Liver biopsy with electron microscopy (EM) can still aid select cases: Byler's bile supports PFIC1, absent bile salt export pump staining suggests PFIC2, and loss of MDR3 indicates PFIC3 in high-GGT cholestasis. However, EM findings, such as bile duct paucity or absent peroxisomes, are often subtle, or nonspecific, especially in infancy. Given its invasiveness and limited sensitivity, biopsy is best reserved for cases with inconclusive genomic and biochemical testing.

In unresolved cases, broader sequencing approaches such as whole genome sequencing (WGS) with long reads may identify deep intronic or structural variants missed by probe-based panels and short-read exome sequencing. In patients with persistent cholestasis and nondiagnostic panel results, whole exome sequencing or WGS may identify pathogenic variants in genes not included in targeted panels or not yet associated with cholestasis. These approaches provide a comprehensive, hypothesis-free evaluation of the genome and can improve diagnostic yield, particularly in complex or syndromic cases. However, their use should be balanced against considerations of cost, turnaround time, and the increased likelihood of detecting VUS. Accordingly, broader genomic sequencing is best considered as a second-tier diagnostic strategy following negative targeted panel testing.

## 5 Conclusion

Collectively, these findings demonstrate the value of broad, panel-based genetic testing in uncovering the diverse and evolving genetics of pediatric cholestasis. As molecular diagnostics advance, integrating genomic data with clinical, biochemical, and functional evidence will remain essential. Future research should prioritize systematic genotype–phenotype correlation and functional validation to refine variant interpretation. A collaborative, gene-informed approach will be critical to improving diagnostic accuracy, personalizing treatment, and advancing care for children with cholestatic liver disease.

## Data Availability

The datasets presented in this article are not publicly available because the underlying genetic testing data were generated under patient consent and testing agreements that restrict data sharing and public deposition. Requests regarding limited deidentified data may be directed to the corresponding author, subject to institutional approval and applicable consent and privacy restrictions.
